# Silver coating on dental implant-abutment connection screws as potential strategy to prevent loosening and minimizing bacteria adhesion

**DOI:** 10.3389/fbioe.2023.1293582

**Published:** 2024-01-09

**Authors:** Eugenio Velasco-Ortega, Alvaro Jiménez-Guerra, Iván Ortiz-Garcia, Enrique Nuñez-Márquez, Jesús Moreno-Muñoz, Javier Gil, Luis M. Delgado, José Luis Rondón-Romero, Loreto Monsalve-Guil

**Affiliations:** ^1^ Comprehensive Dentistry for Adults and Gerodontology, Master in Implant Dentistry, Faculty of Dentistry, University of Seville, Seville, Spain; ^2^ Bioengineering Institute of Technology, Universitat Internacional de Catalunya, Barcelona, Spain

**Keywords:** silver coating, dental implant, screws loosening, antibacterial coating, tightening

## Abstract

**Introduction:** One of the main problems for the long-term behavior of dental implants are loosening of the implant-abutment connection screws and bacterial infiltration. The aim of this work is to increase the screw fixation by silver coating, providing superior mechanical retaining and antibacterial effect.

**Methods:** Eighty dental implants with their abutments and screws have been studied. Twenty screws were not coated and were used as a control while the rest of screws were silver coated by sputtering, with three different thickness: 10, 20 and 40 μm and 20 screws per each thickness. Coating morphology and thickness were determined by scanning electron microscopy using image analysis systems. The screws were tightened for each of the thicknesses and the control with two torques 15 Ncm and 20 Ncm and tested under mechanical fatigue simulating oral stresses up to a maximum of 500,000 cycles. The remaining torques at different cycles were determined with a high-sensitivity torquemeter. Cell viability assays were performed with SaOs-2 osteoblasts and microbiological studies were performed against *Streptococcus gordonii* and *Enterococcus faecalis* bacteria strains, determining their metabolic activity and viability using live/dead staining.

**Results:** It was observed a decrease in torque as cycles increase. For a preload of 15 Ncm at 100,000 cycles, the loosening was complete and, for 20 Ncm at 500,000 cycles, 85% of torque was lost. The silver coatings retained the torque, especially the one with a thickness of 40 μm, retaining 90% of the initial torque at 500,000 cycles. It was observed that osteoblastic viability values did not reach 70%, which could indicate a slight cytotoxic effect in contact with cells or tissues; however, the screw should not be in direct contact with tissue or living cells. Silver coating induced a significant reduction of the bacteria metabolic activity for *Streptococcus gordonii* and *Enterococcus faecalis*, around 90% and 85% respectively.

**Discussion:** Therefore, this coating may be of interest to prevent loosening of implant systems with a worthy antibacterial response.

## 1 Introduction

**FIGURE 1:**
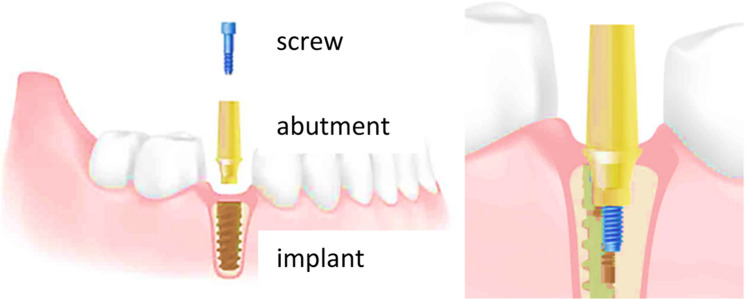
Diagram of the arrangement of the elements that make up a dental implant.

Dental implants are composed of two main parts: the endosseous screw, properly called the implant, which is the piece that will be in contact with the bone; and the emerging part which is the prosthesis, where a crown will be placed. In most cases, this restoration is usually cemented onto the abutment. The abutment and the implant are joined in the great majority of implant systems by a screw that is usually made of Ti-6Al-4V alloy (Adell et al., 1981; Brånemark, 1983). A schematic of a dental implant can be seen in Figure 1.

One of the most frequent causes of dental implant revision is the loosening of the implant-prosthesis connection screw, which causes misalignment between the implant and the prosthesis and, in some cases, it could lead to breakage of the screw with successive chewing loads. This problem causes significant discomfort to the patient, and it is not an easy operation for the surgeon since surgeon must remove the part of the screw inside the core of the osseointegrated implant (Carlson and Carlsson, 1994; Goodacre et al., 1999; Lekholm et al., 1999; Calderon et al., 2014; Lee et al., 2020). The screw thread is sometimes deformed and, in consequence, it may be necessary to remove the implant and place a new one of larger diameter, if the bone and bone space allow it (Zarb and Schmitt, 1990; Al Jabbari et al., 2008; Tsuge and Hagiwara, 2009).

The two main causes of failure of a threaded assembly should be considered: tension relaxation and self-loosening (Adell et al., 1981; Brånemark, 1983; Zarb and Schmitt, 1990; Carlson and Carlsson, 1994; McGlumphy et al., 1998; Goodacre et al., 1999; Lekholm et al., 1999; Winkler et al., 2003; Al Jabbari et al., 2008; Tsuge and Hagiwara, 2009; Calderon et al., 2014; Lee et al., 2020). Regarding the tension relaxation, a threaded assembly “relaxes” when there is a permanent change in the axial length of the screw, when the substrate itself relaxes, as in sealing joints, or when the temperature changes. This phenomenon reduces the bolt tension and reduces the residual clamping force. Permanent length changes can occur due to settling rough faces of adjacent parts (e.g., nuts and washers soften under the pressure exerted by the bolt) and permanent deformation (the surface pressure on the bearing face of the bolt or nut exceeds the compressive strength of the stressed part material) (Adell et al., 1981; Brånemark, 1983). To avoid stress relaxation the elasticity of the assembly can be increased so that the expected degree of settlement and permanent deformation can be compensated for, a decrease in the pre-stressing force can be largely avoided. This is possible with screws with a high l/d ratio (l = length, d = diameter), nuts and bolts with flange on the head, thus washers that reduce the surface pressure and, therefore, the seating on the bearing surfaces, nuts and bolts with elastic washer embedded in the head or with concave support washers or conical spring washers or concave springs (Winkler et al., 2003).

Regarding self-loosening, the clamping load is maintained by the preload of the bolt after tightening. This occurs because the bolt has been stretched like a spring and the tension acts by pulling the nut towards the head and thus compressing the fastened elements. Once the clamping force is removed, the tension acts by loosening the nut from the bolt. The friction of the threads and under the head of the bolt and nut opposes this force and maintains the bolt tension (Siamos et al., 2002; Kim et al., 2003). If the system being fastened is subjected to alternating loads or vibration the locking effect caused by the friction components decreases, so the nut rotates on the thread releasing tension. Vibrations can be longitudinal, transverse or combined. Transverse vibrations due to alternating horizontal loads are much more damaging and can quickly loosen a normal untreated threaded element. Longitudinal forces due to pulsating axial loads also cause lodging, although to a lesser degree.

High tensile strength bolts and screw designing can prevent uncontrolled loosening of properly loaded bolts. The use of high tensile strength bolts allows higher pre-stressing forces, which are sufficient to prevent relative movement. A design that increases the l/d ratio increases the elasticity of the assembly. Historically, a ratio of l/d > 6 has been considered optimal (McGlumphy et al., 1998). Friction can be increased by modifying the surface finish and structure of the bolt and nut bearing surfaces. By applying adhesive, the degree of freedom from lateral movements is eliminated due to the fact that the clearances are completely filled and, at the same time, thread friction due to the interfacial connection once the adhesive has cured. By creating a tight connection, i.e., bolts integrated into the part, spot welds, thread slippage can be limited (McGlumphy et al., 1998). These design concepts should be applied to the standard dental implant applications.

In the field of Dentistry, clinicians typically employ screws for one specific purpose and with very few exceptions: to generate clamping force. To avoid movement between the implant and abutment it is necessary that the screw is very well fixed to the two components in the long term. Improved fixation will prevent misalignments that can lead to screw fracture as well as bacterial leakage. In addition, there may come appointments where clinicians need to disassemble the system and prosthetic screws perform this function well, but their technical design must be precise to ensure satisfactory and long-term outcomes.

To secure an assembly, clinicians apply torque through screw head. A clockwise torque reduces the gap between the screw head and the prosthesis. If resistance is encountered, as when fastening a flange, the screw will persistently rotate until equilibrium is reached between the torque applied to the head and the reaction torque of the assembly. Two components contribute to this reaction torque: friction between the coincident threads and a vertical load induced by the screw torque (Siamos et al., 2002; Kim et al., 2003; Cicciù et al., 2019; Cervino et al., 2022).

The equilibrium relationship is usually expressed, mathematically, by this Eq. 1:

T=K·d·F (1)

Where T is the torque, d is the screw diameter, F is the clamping load, and K is an empirical constant, which considers friction and the variable diameter under the head and in the threads where friction acts.

Different screw designs and fastening systems have been studied, albeit with limited success (Boggan et al., 1999; Lang et al., 1999; Michalakis et al., 2003; Shin et al., 2014). One potential approach to increase the fixation of dental implant screws is to manufacture screws using very ductile alloy, such as precious metals. Nobel Biocare used gold screws, which achieved high fixation due to increased friction through plastic deformation of the screw (Alsubaiy, 2020; Selvamani et al., 2022). However, this type of screw had two problems: the difficulty of extracting the gold screw, rendering implant revisions impractical, and the high economic cost. In this research, we attempt to address this issue by applying a silver coating onto Ti6Al4V screws; Ti6Al4V alloy will guarantee a good mechanical resistance, while silver coating will enhance screw fixation due to the great plastic deformation of silver.

Furthermore, silver is well-established for its bactericidal properties (Godoy-Gallardo et al., 2014; Godoy-Gallardo et al., 2015; Boutinguiza et al., 2018; Fernández-Arias et al., 2019; Ghiuta and Cristea, 2020). This screw coating will prevent bacterial infiltration at the implant-abutment connection, providing an additional benefit in preventing peri-implantitis. Numerous studies have explored the bactericidal effect of silver in nanoparticle form since silver nanoparticles do not oxidize and prevent the formation of gingival tissue staining (Godoy-Gallardo et al., 2016a; Godoy-Gallardo et al., 2016b). It is worth noting that the connection is not in contact with the gingiva and, therefore, the possible formation of black oxides should not affect soft tissue or lead to undesirable aesthetic effects.

The objective of this research is to reduce the loosening of the screw that fixes the dental implant and the abutment by means of silver coatings of different thicknesses on the connection screw. In addition, the cytocompatibility of the coating and the bacteria response are studied using two types of strains usuals in the oral cavity: *Streptococcus gordonii* and *Enterococcus faecalis.*

## 2 Materials and methods

### 2.1 Material preparation

Eighty IPX^®^ dental implant system complete: dental implants (ICI04012) with diameter 4 mm and length of 12 mm, screws (TP4048) diameter 2 mm and abutments RISB40 diameter 4 mm kindly donated by the Galimplant (Galimplant, Spain) were used. Twenty Ti6Al4V screws were not silver coated and were used as control. Sixty screws were subjected to silver sputtering at different times 5, 12, and 27 min to achieve coatings of 10, 20 and 40 μm, twenty units per studied thickness. Prior to sputtering, a vacuum is created in the chamber and Ar 5.0 is introduced at room temperature. The silver film used is 99.99% pure. For biological and microbiological tests, eighty discs of the same Ti6Al4V of the screws were used. The discs measured 5 mm in diameter.

### 2.2 Coating analysis

The samples were cut transversely with a diamond disc cutting machine (Microstruers X208 Danmark), at a very slow speed of 10 rpm in the presence of a lubricant composed of a mixture of ethyl and methyl alcohol. The samples were dried by gas flow. The cuts were observed using scanning electron microscopy (JEOL 6400, Japan) and the silver thickness of each series of screws was determined. For the determination of the thickness, the image analysis program (ImageJ, NIH, United States) was used. The thickness of the silver was measured by Field Emission Scanning Electron Microscope (ThermoFisher, Quattro ESEM) using nitrogen ion beam.

### 2.3 Masticatory forces and loosening behavior simulation

The prosthetic abutments were connected to the dental implant and screws were tightened by means of a high sensitivity (±0.1 Ncm) torque meter (Tonishi, Japan), using two different torques: 15 and 20 Ncm.

**FIGURE 2:**
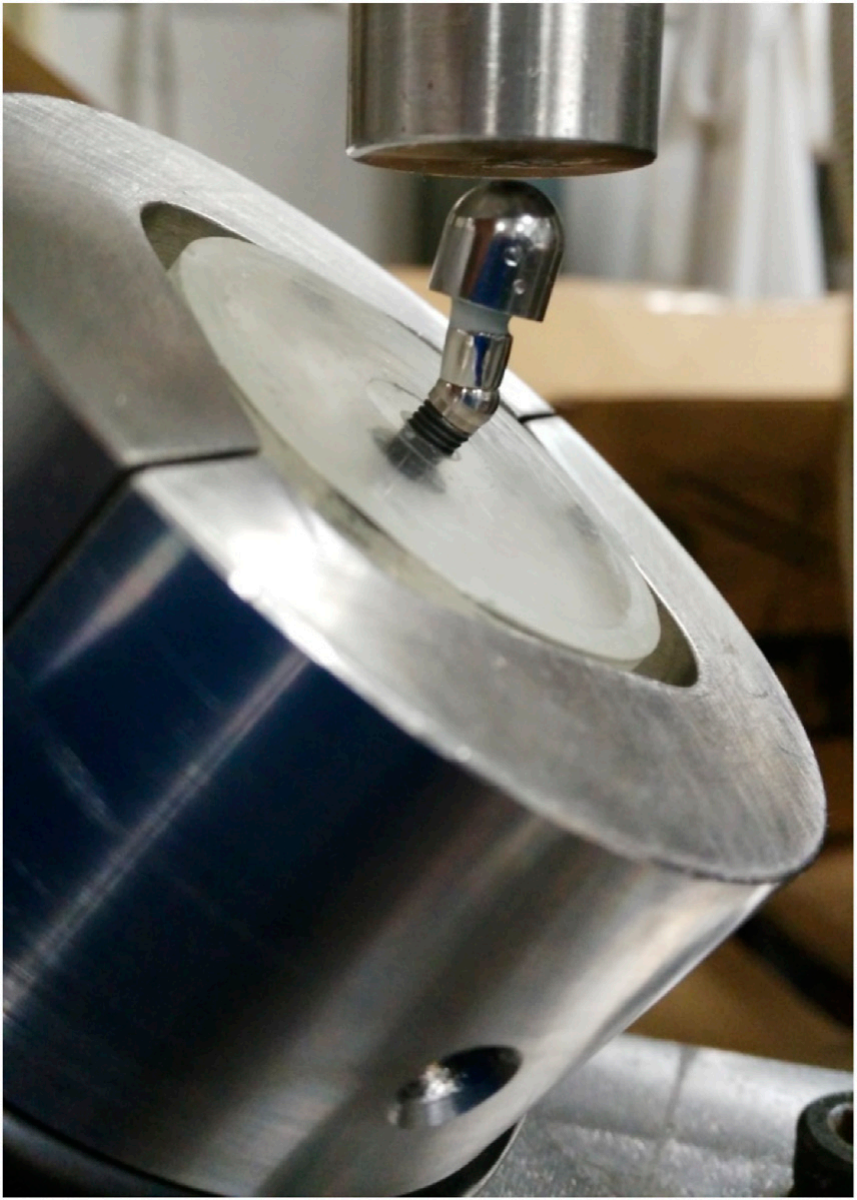
Mechanical tests at 30° according to the standard ISO 14801.

The loosening behavior of dental implants due to chewing cycles was determined by means of hydraulic testing machine (Bionix, MTS, United States) and a chamber at 37°C. The systems were placed within a resin at 30° relative to the vertical and the bending loads were applied. The dental implant system and assembly are shown in Figure 2. Test was performed according to the ISO 14801 standards for fatigue testing of endosseous implants (Gil et al., 2012; International Organization for Standardization, 2016). The 30° is the maximum angle recommended for placement of a dental implant in the mouth (Schwartz-Arad et al., 2005; Gil et al., 2009; Gil et al., 2014). The main testing parameters were compression load from 39.2 to 392.4 N combined with a 3° torsion and using a triangular function, 10 Hz frequency and up to 500,000 cycles.

### 2.4 Screw loosening determination

At 500,000 cycles, the test was stopped and the torque of the connection screw between the dental implant and the sleeve was determined by means of a torque meter (Tohnichi, Japan).

### 2.5 Cell response

An osteoblastic cell line (SaOs-2; ATCC, United States) was used for the *in vitro* assays. SaOs-2 cells were cultured using McCoy’s modified 5A medium, supplemented with 10% fetal bovine serum (FBS, Gibco, United States), 1% penicillin/streptomycin (Invitrogen, United States), and 1% sodium pyruvate (Invitrogen, United States). Cultures were grown at 37 °C, 5% CO_2_ under humidified conditions.

Cytocompatibility studies are only performed on the 40 μm thick silver and control samples since it is a surface property, and it is not necessary to perform them with the other thicknesses since in all cases the area in contact will be the same.

Confluent cells were detached with trypsin (Invitrogen, Carlsbad, CA, United States) for 5 min. A cell density of 5,000 cells per sample was seeded on each disc and incubated at 37°C for 3 and 21 days. At each time point, samples (n = 5) were washed with PBS and moved onto a new plate to perform the metabolic activity assay using Alamar Blue (Invitrogen-Thermo Fisher Scientific, United States), following manufacturer instructions. Briefly, 10% Alamar Blue in media was incubated in direct contact with for 4 h at 37°C. Wells without cells were used as a blank. Uncoated samples were used as 100% metabolic activity at each time point.

### 2.6 Bacterial adhesion

For the adhesion assays, two different bacteria strains, *Streptococcus gordonii* (CECT 804) and *Enterococcus faecalis* (CECT 795) were studied, cultured in tryptic soy broth (TSB) and brain heart infusion (BHI) for *S. gordonii* and *E. faecalis*, respectively. Bacteria inoculums were prepared by resuspending two bacteria colonies in 5 mL media and growing overnight at 37°C. Subsequently, bacteria suspension was diluted to an optical density of 0.2 at 600 nm.

Specimens (n = 5) were sterilized by immersion in 70% ethanol for 15 min and then, washed with sterile saline to avoid ethanol cross effect. Sterile samples were placed in 24-well plates and 700 µL of diluted bacterial suspension was also placed into. Samples were incubated at 37°C for 2 h and, after this incubation time, bacterial adhesion was analyzed by washing once with PBS and then, samples were transferred to a clean 24-well plate for metabolic and live–dead assays. An empty well plate was used as a positive control of bacteria growth for the metabolic activity assay.

For the metabolic activity assays, samples and controls were incubated with 650 µL resazurin at 25 μg/mL in PBS (Sigma-Aldrich, United States) until positive control was saturated. Then, 100 µL was transferred to a 96-well plate and the absorbance was read at 570 and 600 nm. Positive control was used as a 100% metabolic activity reference.

For the bacteria viability assay, samples were stained with LIVE/DEAD^®^ BackLight™ Bacterial Viability Kit solution (Thermo Fisher Scientific, United States), as per manufacturer instructions. Briefly, SYTO and propidium iodide reagents were diluted in a proportion of 1.5 µL per mL of PBS, and samples were incubated with 650 µL of the solution for 15 min at 37°C. Then, samples were washed twice with PBS and samples were visualized at three different regions using a confocal microscope at ×64 (DMI8, Leica, Germany) using 589/615 nm excitation/emission wavelengths for dead cells and 495/520 nm, for live cells.

### 2.7 Statistical analysis

The number of samples used was obtained by an experimental sample size method. Statistical analysis was performed using MiniTab 17 software (Minitab Inc.). Kruskal–Wallis and Mann Whitney U non-parametric tests were used to compare the different conditions to each other. Statistical differences were considered with *p* < 0.001.

## 3 Results

### 3.1 Coating analysis

**FIGURE 3:**
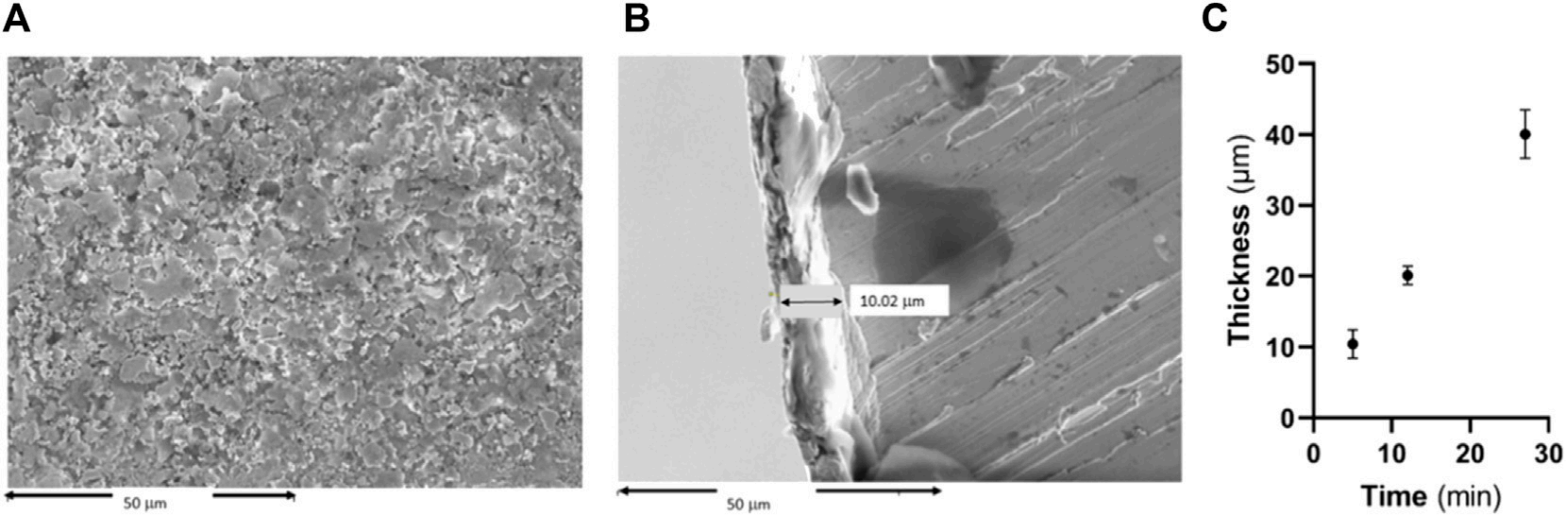
**(A)** Surface of the screw coated by silver, **(B)** cross section of the silver-coated screws and **(C)** Measurement of the thickness of silver coating.

In Figures 3A–C, it can be observed the surface obtained by silver sputter and the measurement of the thickness, showing relatively rough surfaces. In order to adjust the coating thickness, the required time to obtain 10, 20 and 40 µm coating was studied, being 5, 12 and 27 min, respectively. The silver deposition started at a ratio of 2.05 μm/min during the initial 5 min and finished being under 1.33 μm/min for the longer times.

### 3.2 Screw loosening determination

**FIGURE 4:**
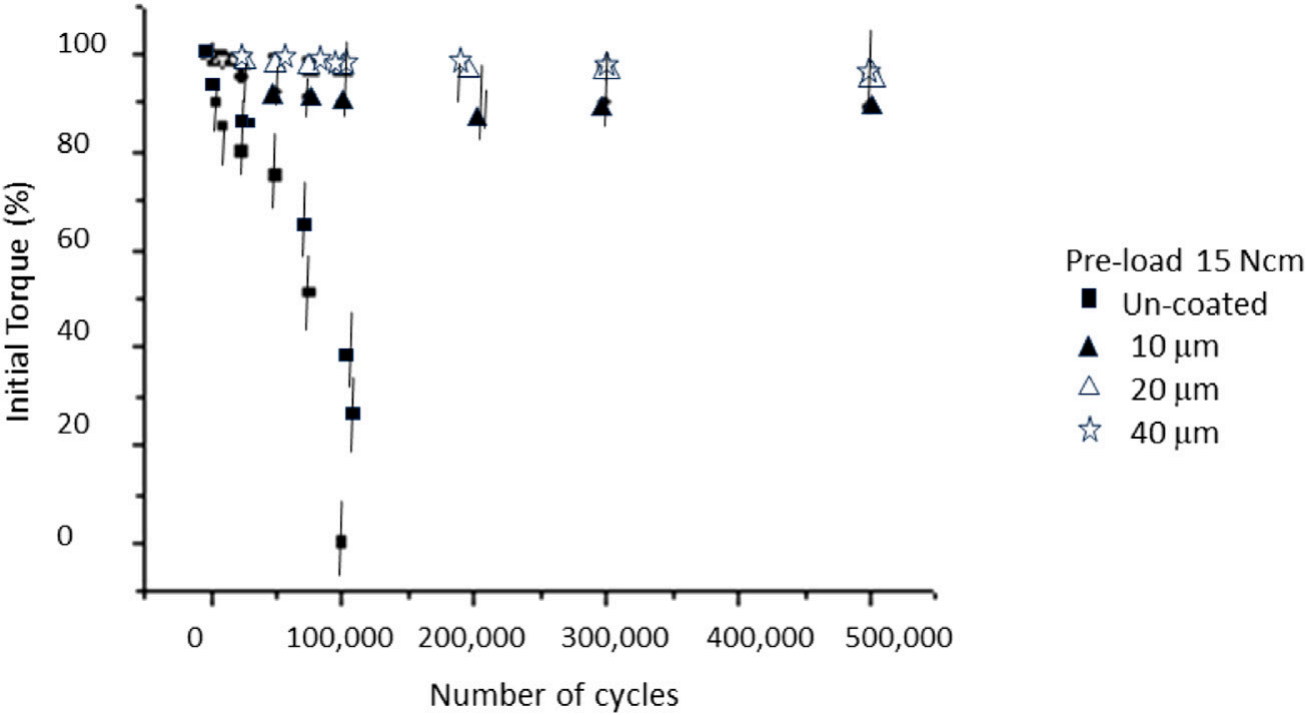
Loosening of the initial torque with the number of cycles for a pre-load of 15 Ncm. Statistical difference significance was un-coated in relation to the 10, 20 and 40 μm with *p* < 0.001.

**FIGURE 5:**
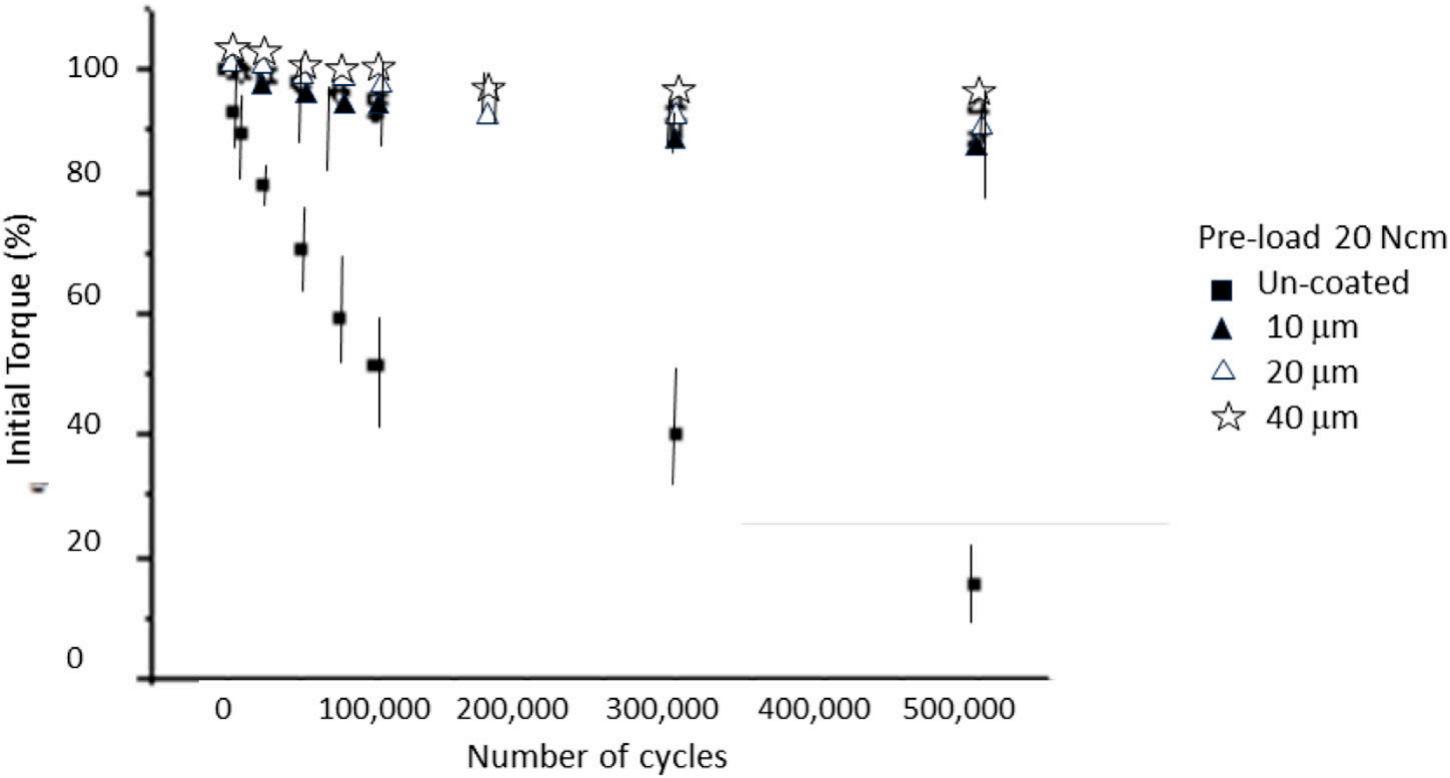
Loosening of the initial torque with the number of cycles for a pre-load of 20 Ncm. Statistical difference significance was un-coated in relation to the 10, 20 and 40 μm with *p* < 0.001.

In Figures 4, 5, it can be seen the graphs representing the percentage of loss of tightening with respect to the initial torque of 15 Ncm and 20 Ncm, respectively.

In the case of the non-coated case, a very significant loosening can be observed after the first mechanical cycles. The silver coated screws had a smaller loosening, corresponding to a smaller loosening with a silver thickness of 40 µm. It can be seen in Figure 5 that increasing the tightening torque to 20 Ncm causes the loosening rate to be lower than the initial torque of 15 Ncm. From Figures 4, 5, we can observe that the thickness of the silver layer did not present a statistically significant change in loosening with a *p* < 0.001.

### 3.3 Cell response

**FIGURE 6:**
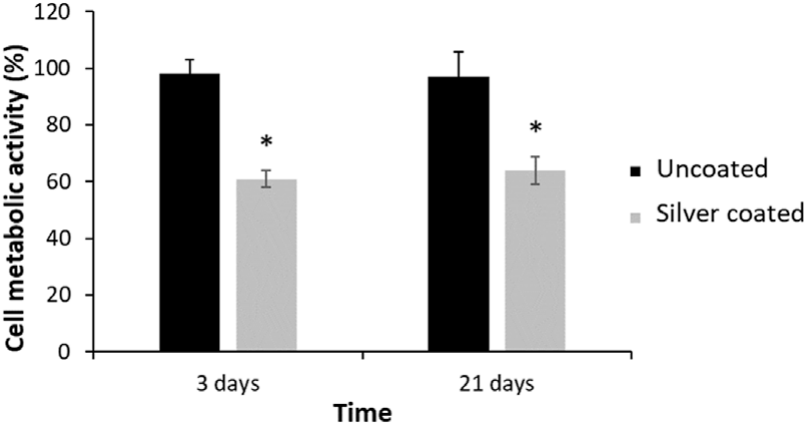
SaOs-2 osteoblastic cells metabolic activity for the Ti6Al4V, as control, and the silver coated surfaces at 3 and 21 days. Metabolic activity was normalized against Ti6Al4V at each time point. Statistical differences are represented with * (*p* < 0.001).

Figure 6 shows the cell metabolic activity at days 3 and 21 using osteoblastic cells. The metabolic activity has been obtained by determining the AlamarBlue reduction, being proportional to the cell viability on the different surface types and it was normalized against Ti6Al4V for each time point. It can be seen that after both 3 and 21 days of culture, there is a significant difference with *p* < 0.001 on the number of cells between uncoated and silver coated.

### 3.4 Early bacterial adhesion

**FIGURE 7:**
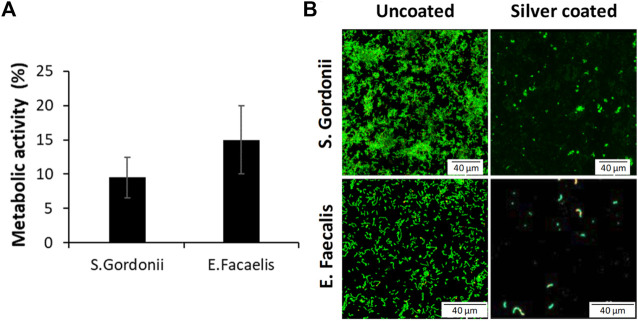
**(A)** Reduction of the metabolic activity of silver coated samples and **(B)** Live-Dead staining against *Streptococcus gordonii* and *Enterococcus faecalis*.

The results of bacteria metabolic activity and viability assays for each bacteria strain after 2 h of direct contact are represented in Figure 7. There was significant reduction of the bacteria metabolic activity for *Streptococcus gordonii* and *Enterococcus faecalis*, when compared to the uncoated controls. This evidence was confirmed by the Live–Dead staining, where an important reduction of both adhered bacteria was observed on the silver coated surfaces in relation to the uncoated ones.

## 4 Discussion

In this work, we studied the impact of different thicknesses of silver coatings on the tightening torque for the connection due to the cyclic masticatory forces. The fatigue tests realized have been carried out with the same stress triaxiality as in the mouth (tension-compression and torsion).

For uncoated screws, the average initial torque conservation (15 Ncm) remained at 79.9% after 25,000 cycles. This retention decreased to 75% after 50,000 cycles, further reduction up to 50% was observed after 75,000 cycles and screws were completely loosened after 100,000 cycles. The loosening results for screws with a torque of 20 Ncm decreased the initial torque to values of 15% of the original with 500,000 cycles. The loosening results between an initial torque of 15 Ncm and 20 Ncm show statistically significant differences with a *p* < 0.001.

As can be seen in Figures 4, 5, these results already give us a glimpse of considerable loosening of the implants. In a typical scenario, chewing load amounts to approximately 30 kg and the loosening process could occur over 500,000 cycles, around 14 months. However, for patients who exhibit bruxism, this timeline may reduce to just 5 months. Therefore, it is essential for clinicians to monitor dental implants and perform regular screw tightening, as emphasized in references (Robinson et al., 2019; Dorado et al., 2022).

Regarding silver as a coating material, it possesses a high plasticity, leading to an increase in friction between the screw and the implant core and, consequently, increasing the retention. Silver has a lower modulus of elasticity (E = 69 GPa) compared to titanium (E = 110 GPa). This lower modulus of elasticity and higher deformability of silver promotes a snug fit between the screw and the thread, which causes a larger section of screw-thread contact area between them. In turn, this expanded contact area enhances friction forces, leading to a more secure screw fixation.

Figure 4 shows the values obtained for the silver-coated screws when preloaded with a torque of 15 Ncm and Figure 5 for 20 Ncm. The results showed that for screws with different coating thicknesses there are statistically significant differences (ANOVA *p* < 0.001) between those of 10 μm and 20 and 40 µm. The thicknesses of 20 and 40 µm showed a better retention, this fact means that 20 µm was a saturation value; in other words, even if the thickness is increased, there would not be any benefit in retention (Nicolas-Silvente et al., 2020). The values obtained were close to 90% of the initial torque when screws were subjected to 500,000 cycles for 20 µm coating, and 80% for 10 µm.

There are no statistically significant differences in the values of percentage of loosening when the preloads are different, at least between those studied, which were 15 and 20 Ncm. However, a great improvement in retention was observed when comparing these retention values with the silver coated screws with those that do not present this layer, varying from 90% retention to 60% retention of the non-coated screws.

Cell response demonstrated a reduction of metabolic activity of osteoblasts, being between 60% and 65%. These values are lower than the 70% indicated by the cytocompatibility standard. This fact shows that the silver coating treatment is slightly cytotoxic (International Organization for Standardization, 2009; Velasco-Ortega et al., 2016; Velasco-Ortega et al., 2020). However, if a material does not meet this requirement, it can be implanted in the human body if the benefit is much higher than the detrimental effect. Moreover, in our case, this result should not be of concern since the prosthetic screw coated with silver corresponds to the connection between the implant and the abutment and, in principle, should not be in contact with human cells. In the case that this coating would be in contact with cells, more exhaustive studies should be carried out to determine its behavior and ensure its biocompatibility. Nor should there be any concern about soft tissue discoloration caused by the silver that forms tattoos and compromises the esthetics of the implant since the screw is not in contact with the gingiva (Rawal et al., 2007; Brooks et al., 2020; Velasco-Ortega et al., 2021).

Regarding the bacteria response, it has been possible to verify the reduction of bacteria adhered to the silver coated surfaces for two bacteria strains (*Streptococcus gordonii* and *Enterococcus faecalis*) common in the oral cavity and in the bacterial filtrations in the implant-abutment systems. These antibacterial strategies are very important since a suction of the oral cavity fluids occurs in the gap between the dental implant and the abutment. This suction of saliva transports different types of bacteria that in contact with the silver coated area will inhibit the possibility of biofilm formation or that these bacteria are deposited on the surface of the dental implant. Therefore, this silver layer that produces a better fixation between the implant and the abutment also has a detrimental effect on bacteria metabolic activity that can contribute to avoid perimplant related diseases (Berglundh et al., 2018; Rakic et al., 2018; Schwarz et al., 2018). One of the future studies of this research would be the study of the action of the silver coating when we have biofilm. In this work, two bacteria strains have been used with good results but its action in a biofilm with the most common bacteria in peri-implantitis disease should be checked.

This work has certain limitations since we had to simulate chewing cycles at a certain frequency. However, it is important to recognize that each individual patient may have a unique chewing pattern that cannot be precisely replicated. Additionally, the food and beverage consumption may be a factor to be considered, although we believe its impact on our results would be minimal. For future research, it would be advisable to conduct trials involving different bacterial strains against this type of silver coating to gain a more precise understanding of the antibacterial capacity across the entire spectrum of oral cavity bacteria. It is necessary the study of the bacteria adhesion and the antibacterial effect of the silver coating at short and long term.

## 5 Conclusion

The silver coating reduced loosening of the connecting screws between the dental implant and the abutment. Silver coating maintains 90% of the initial tightening torque after 500,000 cycles for the two pre-loads studied, 15 and 20 Ncm. The thickness that achieves the greatest fixation in the implant system corresponds to that of 20 and 40 µm. It has been possible to determine a reduced osteoblastic metabolic activity of the coating, but in principle the silver will not be in contact with living tissue. A reduction of metabolic activity of *S. gordonii* and *E. faecalis* around 10% and 15% respectively has been determined. This coating may be of interest for its application in the dental clinic as an anchorage for screws and for its potential antibacterial capacity.

## Data Availability

The original contributions presented in the study are included in the article/Supplementary material, further inquiries can be directed to the corresponding authors.
